# Comparative Effectiveness of a Technology-Facilitated Depression Care Management Model in Safety-Net Primary Care Patients With Type 2 Diabetes: 6-Month Outcomes of a Large Clinical Trial

**DOI:** 10.2196/jmir.7692

**Published:** 2018-04-23

**Authors:** Shinyi Wu, Kathleen Ell, Haomiao Jin, Irene Vidyanti, Chih-Ping Chou, Pey-Jiuan Lee, Sandra Gross-Schulman, Laura Myerchin Sklaroff, David Belson, Arthur M Nezu, Joel Hay, Chien-Ju Wang, Geoffrey Scheib, Paul Di Capua, Caitlin Hawkins, Pai Liu, Magaly Ramirez, Brian W Wu, Mark Richman, Caitlin Myers, Davin Agustines, Robert Dasher, Alex Kopelowicz, Joseph Allevato, Mike Roybal, Eli Ipp, Uzma Haider, Sharon Graham, Vahid Mahabadi, Jeffrey Guterman

**Affiliations:** ^1^ Suzanne Dworak-Peck School of Social Work University of Southern California Los Angeles, CA United States; ^2^ Roybal Institute on Aging University of Southern California Los Angeles, CA United States; ^3^ Daniel J. Epstein Department of Industrial and Systems Engineering Viterbi School of Engineering University of Southern California Los Angeles, CA United States; ^4^ Schaeffer Center for Health Policy and Economics University of Southern California Los Angeles, CA United States; ^5^ Policy Analysis Unit Los Angeles County Department of Public Health Los Angeles, CA United States; ^6^ Keck School of Medicine University of Southern California Los Angeles, CA United States; ^7^ Los Angeles County Department of Health Services Los Angeles, CA United States; ^8^ College of Social and Behavioral Sciences California State University, Northridge Los Angeles, CA United States; ^9^ Department of Psychology Drexel University Philadelphia, PA United States; ^10^ Caremore Medical Group East Haven, CT United States; ^11^ Herbert Wertheim College of Medicine Florida International University Miami, FL United States; ^12^ Department of Health Policy and Management Fielding School of Public Health University of California Los Angeles Los Angeles, CA United States; ^13^ Department of Emergency Medicine Northwell Health Long Island Jewish Medical Center New Hyde Park, NY United States; ^14^ David Geffen School of Medicine University of California Los Angeles Los Angeles, CA United States; ^15^ Harbor-UCLA Medical Center University of California Los Angeles Los Angeles, CA United States; ^16^ Los Angeles Biomedical Research Institute Los Angeles, CA United States

**Keywords:** primary care, disease management, depression, diabetes mellitus, health information technology, telemedicine, comparative effectiveness research, propensity score, population health, patient reported outcome measures

## Abstract

**Background:**

Comorbid depression is a significant challenge for safety-net primary care systems. Team-based collaborative depression care is effective, but complex system factors in safety-net organizations impede adoption and result in persistent disparities in outcomes. Diabetes-Depression Care-management Adoption Trial (DCAT) evaluated whether depression care could be significantly improved by harnessing information and communication technologies to automate routine screening and monitoring of patient symptoms and treatment adherence and allow timely communication with providers.

**Objective:**

The aim of this study was to compare 6-month outcomes of a technology-facilitated care model with a usual care model and a supported care model that involved team-based collaborative depression care for safety-net primary care adult patients with type 2 diabetes.

**Methods:**

DCAT is a translational study in collaboration with Los Angeles County Department of Health Services, the second largest safety-net care system in the United States. A comparative effectiveness study with quasi-experimental design was conducted in three groups of adult patients with type 2 diabetes to compare three delivery models: usual care, supported care, and technology-facilitated care. Six-month outcomes included depression and diabetes care measures and patient-reported outcomes. Comparative treatment effects were estimated by linear or logistic regression models that used generalized propensity scores to adjust for sampling bias inherent in the nonrandomized design.

**Results:**

DCAT enrolled 1406 patients (484 in usual care, 480 in supported care, and 442 in technology-facilitated care), most of whom were Hispanic or Latino and female. Compared with usual care, both the supported care and technology-facilitated care groups were associated with significant reduction in depressive symptoms measured by scores on the 9-item Patient Health Questionnaire (least squares estimate, LSE: usual care=6.35, supported care=5.05, technology-facilitated care=5.16; *P* value: supported care vs usual care=.02, technology-facilitated care vs usual care=.02); decreased prevalence of major depression (odds ratio, OR: supported care vs usual care=0.45, technology-facilitated care vs usual care=0.33; *P* value: supported care vs usual care=.02, technology-facilitated care vs usual care=.007); and reduced functional disability as measured by Sheehan Disability Scale scores (LSE: usual care=3.21, supported care=2.61, technology-facilitated care=2.59; *P* value: supported care vs usual care=.04, technology-facilitated care vs usual care=.03). Technology-facilitated care was significantly associated with depression remission (technology-facilitated care vs usual care: OR=2.98, *P*=.04); increased satisfaction with care for emotional problems among depressed patients (LSE: usual care=3.20, technology-facilitated care=3.70; *P*=.05); reduced total cholesterol level (LSE: usual care=176.40, technology-facilitated care=160.46; *P*=.01); improved satisfaction with diabetes care (LSE: usual care=4.01, technology-facilitated care=4.20; *P*=.05); and increased odds of taking an glycated hemoglobin test (technology-facilitated care vs usual care: OR=3.40, *P*<.001).

**Conclusions:**

Both the technology-facilitated care and supported care delivery models showed potential to improve 6-month depression and functional disability outcomes. The technology-facilitated care model has a greater likelihood to improve depression remission, patient satisfaction, and diabetes care quality.

## Introduction

### Depression Care in Underserved Populations

Depression, an underdiagnosed comorbidity for those with chronic illness [1], impairs functional status and worsens clinical outcomes, including morbidity and mortality; it also increases cost [2-5]. Diabetes doubles the risk of depression relative to the general population; 10% to 15% of adults with diabetes also have major depressive disorder [6,7]. The relationship between diabetes and depression might be bidirectional [8,9]. Depression with diabetes may significantly worsen the course of both disorders, leading to reduced functioning and quality of life [8-11].

Low-income, culturally diverse populations with chronic illnesses have an even higher risk of depression [7,12,13]. Hispanics and Latinos have a higher prevalence of diabetes compared with non-Hispanic whites [14] and are less likely to meet glycated hemoglobin (HBA_1c_) and serum cholesterol goals [15]. Racial and ethnic minority populations also experience disparities in terms of mental health care, including appropriate mental health diagnosis, counseling, antidepressant medication prescriptions, and depression care follow-up [16-23]. Hispanics and Latinos are less than half as likely as non-Hispanic whites to receive guideline-level depression care [20].

Research has shown that there are effective ways to reduce these disparities. For example, there is growing evidence that a team-based collaborative depression care model is effective in improving care for low-income patients (including racial and ethnic minority populations) with chronic illnesses [24-27]. The US Preventive Services Task Force recommends depression screening, and an adaptive treatment approach has been shown to be effective in helping patients find successful antidepressant options or psychotherapy [28,29].

Safety-net primary care clinics are the preferred venue for underserved patients to access depression care because it is a common point of service delivery [30-32]. However, these settings encounter a complex mix of patient, provider, and health system factors that can impede the adoption of evidence-based collaborative depression care and result in persistent disparities in depression outcomes [33].

Safety-net systems organize and deliver a significant level of health care and other related services to uninsured, Medicaid, and other vulnerable populations [34]. Safety-net primary care providers often find it challenging to engage patients with major depression, particularly when it is accompanied by chronic illnesses, because the patient must participate in active and ongoing depression symptom assessment and management in addition to managing the other medical conditions [16,33,35-37]. Concurrently, minority patients are less likely to voluntarily report depressive symptoms, may view depression as a moral weakness or character flaw rather than an illness, and may be more likely to ascribe symptoms of depression to a physical illness [33,38]. Therefore, low-income, predominantly minority patients in safety-net care systems often miss out on depression diagnosis and follow-up assessments [33,39].

### Diabetes-Depression Care-Management Adoption Trial

The Diabetes-Depression Care-management Adoption Trial (DCAT) is a translational study in a large safety-net system of primary care settings. The study compared a technology-facilitated care (TC) model with a usual care (UC) model and a supported care (SC) model to assess whether technology could facilitate the adoption of collaborative depression care for patients with type 2 diabetes. The DCAT TC model is an information and communication technology (ICT)–facilitated care management approach that harnesses automated telephone assessment (ATA) technology and integrates it with a disease management registry (DMR) system to automate key aspects of depression care. The UC model is standard care in a safety-net system in which primary care physicians (PCPs) develop individualized treatment plans for depression and diabetes care. The SC model is a team-based collaborative care management approach that involves care team staff members to provide depression and diabetes care.

The DCAT study is expected to fill two important gaps in current collaborative depression care implementation research. First, existing studies largely rely on labor-intensive, team-based SC approaches to implement collaborative depression care [24-27,40-43]. There is evidence that this SC model is effective and can be cost-effective compared with UC [24-27,44-47]. However, it is challenging for SC teams to integrate depression comorbidity care when patients are presented with other medical conditions because of the intensive labor and time needed to proactively screen for depression, follow up on treatment, and monitor and manage long-term care [33,41,48]. By relieving providers in resource-constrained safety-net clinics from many labor-intensive tasks such as collecting, summarizing, and reviewing individual or aggregate patient data to facilitate care, automation can facilitate the adoption of collaborative depression care. Therefore, DCAT tested ICT that automated critical information collection and processing for depression care tasks, including (1) Depression assessments and symptom monitoring, (2) Patient self-management behavior prompting, (3) Optimization of treatment follow-up, and (4) provider collaborative communication.

Second, existing research has not fully addressed ways to develop a patient-centered approach to implement collaborative depression care for low-income, predominantly minority safety-net patient populations. Prior studies have demonstrated that the team-based approach can effectively implement collaborative depression care in safety-net environments [24-27]; DCAT built on this evidence by applying the ICT to further address language, time, and stigma barriers [33,41,48] affecting safety-net patients. DCAT accomplished this by customizing calls with the patient’s preferred language and call time, making multiple attempts (if needed) to connect with the patient, and establishing a private and machine-only venue to report sensitive depression measures to reduce social desirability bias [49-51]. About 80% of the patients agreed or strongly agreed that the DCAT-tested ICT was easy to use (86.2%, 94/109), nonintrusive (87.1%, 95/109), and private and secure (75.9%, 82/108) [51].

### Paper Purpose and Hypothesis

DCAT-related improvements have been reported in several publications, including trial design [33], TC technology design and evaluation [52], patient acceptance of the technology [51], patient engagement [50], provider implementation reactions [53], depression risk profiling and prediction [54,55], and cost-effectiveness analysis (unpublished data, 2018, [56]). This paper reports DCAT-related depression symptom and diabetes care outcomes after 6 months. In addition, to provide the patients’ perspective on treatment benefits, this paper includes patient-reported outcomes, including physical and mental well-being, functional impairment, and satisfaction with care [57].

The main hypothesis of the paper is that, compared with the UC group, both the TC and SC groups will have statistically significant greater improvement in depression symptoms, diabetes care processes and outcomes, and patient-reported outcomes. TC uses an ATA system to ease the adoption of collaborative depression care rather than direct clinical intervention with patients; therefore, although the researchers have no hypothesis on how TC outcomes will compare with SC outcomes, this paper also explores whether the TC group will have better outcomes than the SC group.

## Methods

### Diabetes-Depression Care-Management Adoption Trial Study Design

DCAT is a translational study conducted in collaboration with Los Angeles County Department of Health Services (LACDHS), the second largest safety-net care system in the United States. Institutional review board approval was obtained from the University of Southern California, the Olive View–University of California Los Angeles Medical Center, and the Los Angeles Biomedical Research Institute.

#### Study Sites and Intervention Period

The study used a quasi-experimental comparative effectiveness design to compare three delivery models in three groups: UC, SC, and TC. Eight clinics were selected to participate in the study based on criteria that reflected geographic and diabetes care model diversity. These clinics were matched by geographic location and patient sociodemographics to form the three study groups. The patients were not randomly assigned; each patient was assigned to a study group based on the clinic from which he or she was recruited.

The UC group featured two community clinics and represents the status quo of clinical practice, wherein the translation and adoption of depression care evidence is performed by PCPs and their staffs. The SC and TC groups each featured two care teams from an LACDHS diabetes disease management program (DMP) to incorporate depression care. In both the SC and TC groups, one of the two teams practiced in both a hospital-based outpatient clinic and a satellite community clinic; the other team practiced in a community clinic in a different geographic area.

The intervention period was 12 months, and the study occurred from 2011 to 2013. During the first 6 months, the UC group received usual primary care, whereas the SC group received DMP-supported depression care, and the TC group received the ATA application in the DMP setting. After 6 months, all SC and TC patients were transferred back to their usual primary care, although the ATA calls were continued for the full 12 months. This paper reports the 6-month outcomes.

#### Intervention Description

Table 1 shows the intervention elements of the UC, SC, and TC models, described below.

**Table 1:** Intervention elements of the usual care (UC), supported care (SC), and technology-facilitated care (TC) models. ATA: automated telephone assessment; DMP: disease management program; DMR: disease management registry; LACDHS: Los Angeles County Department of Health Service; PCP: primary care physicians; PHQ-9: 9-item Patient Health Questionnaire; PST: problem-solving therapy.

Elements	Usual care	Supported care	Technology-facilitated care
Care paradigm	Standard primary care; optional PST	Diabetes DMP-supported care; PST; Interactive DMR system	Diabetes DMP-supported care; PST; ATA linked to DMR enhanced with clinical decision support software
Clinic setting	Two community non-DMP clinics	Two diabetes DMP teams in safety-net clinics: one serving both hospital-based outpatient clinic and a satellite community clinic, and other serving in a community clinic in a different geographic area	Two diabetes DMP teams in safety-net clinics: one serving both hospital-based outpatient clinic and a satellite community clinic, and other serving in a community clinic in a different geographic area
Patient education and care resources	Depression educational pamphlets (in English) or *fotonovella* (in Spanish) adapted for diabetes patients; Standard provider contact and community resource information	Depression educational pamphlets (in English) or *fotonovella* (in Spanish) adapted for diabetes patients; Standard provider contact and community resource information	Depression educational pamphlets (in English) or *fotonovella* (in Spanish) adapted for diabetes patients; Standard provider contact and community resource information
Physician education	Psychiatrist expert conducts webinars about collaborative depression care evidence, offers PCP depression screening and treatment didactic, and provides personal copy of the Los Angeles County Department of Health Services depression care protocol	Psychiatrist expert conducts webinars about collaborative depression care evidence, offers PCP depression screening and treatment didactic, and provides personal copy of the Los Angeles County Department of Health Services depression care protocol	Psychiatrist expert conducts webinars about collaborative depression care evidence, offers PCP depression screening and treatment didactic, and provides personal copy of the Los Angeles County Department of Health Services depression care protocol
Clinician training for PST	Optional for UC clinicians	Mandatory for DMP nurses, nurse practitioners, and social workers; conducted by psychology and social work experts	Mandatory for DMP nurses, nurse practitioners, and social workers; conducted by psychology and social work experts
Depression screen and ongoing symptom monitoring	Standard care determined by PCP practice	Performed by DMP clinical social worker: PHQ-9 screening when patients join the DMP; Ongoing symptom monitoring per clinical judgment based on LACDHS depression care protocol and treatment guideline	Performed by the ATA system and enhanced DMR: Quarterly depression screening (PHQ-2) for nondepressed patients; Monthly symptom monitoring (PHQ-2, -9, other tailored questions) for depressed patients
Depression treatment	Standard care: Antidepressant medication; Referral to clinic social worker or community mental health care	DMP based on the LACDHS protocol and treatment guideline: Antidepressant with optional PST; Option of community referrals	DMP based on the LACDHS protocol and treatment guideline with ATA responses and DMR data: identify at-risk patients, determine treatment, and promptly follow up on treatment adherence issues.
Provider collaborative communication	LACDHS standard clinic collaboration	DMP nurse initiates communication with medication prescriber; Refers patient to social worker if patient refuses medication or needs PST	DMP plus enhanced team care collaboration facilitated by DMR: Reminders prompt designated responders to follow up; Responders include DMP nurse, social worker, medication prescriber or PCP, or psychiatrist
Patient relapse prevention	Standard care	Monthly telephone calls by nurse or social worker	Monthly or quarterly automated telephone calls prompt for relapse prevention.

##### Usual Care Model

The UC model was standard primary care. UC clinicians were offered an optional training opportunity (described in *Provider Training and Depression Treatment Protocol* section below).

##### Supported Care Model

The SC model used the diabetes DMP team (comprising nurse care managers, nurse practitioners, and a consulting or supervising physician) to deliver depression care. SC diabetes care management was designed to proactively identify, risk stratify, and treat patients using clinical protocols that emphasized patient empowerment. The DMP was nurse driven and physician supervised and used structured approaches and protocols; in these programs, nurses delivered the majority of the diabetes care. The approaches included a patient-signed commitment to take an active role in his or her diabetes care, case management, PCP designation, group patient education, self-management support, and care coordination. The diabetes-specific management was provided initially via in-person visits, with follow-up primarily via telephone visits. The DMP included a homegrown, Web-based, interactive chronic DMR system to support clinical assessment and decisions. The DMP was designed for limited-time care management (typically 6 months), after which patients were transferred back to their primary medical providers.

During the study, the SC team supplemented diabetes management with two periodic depression symptom screening and monitoring tools: (1) the 9-item Patient Health Questionnaire (PHQ-9) [58], a standard tool in each clinic’s disease registry and (2) the LACDHS depression care protocol and treatment guideline (see “Provider Training and Depression Treatment Protocol”). In PHQ-9, the patient scores each of the nine Diagnostic and Statistical Manual of Mental Disorders, 4th edition criteria as “0” (not at all) to “3” (nearly every day) to provide both a dichotomous diagnosis of probable major depression and a continuous severity score [58]. The SC program also designated a social worker to provide problem-solving therapy (PST), an evidence-based depression treatment [59].

##### Technology-Facilitated Care Model

The TC model also operated in a DMP clinic setting with a DMR and supplemental depression care based on the LACDHS depression care protocol and treatment guideline. The TC model, however, was designed to assist time-pressured clinical social workers and medical and nursing providers by using an ATA system to routinely screen and monitor patient depression symptoms and treatment adherence and communicate the results to providers. As described elsewhere [33,52], the ATA system was linked with the DMR to automatically trigger depression care management calls on a predetermined calendar schedule. The call contents were individually tailored, driven by a preprogrammed algorithm that scanned patient medical records and call histories to determine applicable questions. The ATA used a persona, “Amy,” who spoke in a natural voice rather than a system-generated text-to-speech robotic voice to administer the assessment questions. During study enrollment, patients selected their preferred language (English or Spanish) and preferred call time. The DCAT ATA built in both automated speech recognition and interactive voice response technologies [60] that allowed patients to either speak their responses to Amy’s questions or punch numbers on a phone keypad. Automated speech recognition has the advantage of eliminating number-punching errors, which are a concern for diabetes patients with sensing or vision problems. Unfortunately, automated speech recognition was only available in English, not Spanish, because of suboptimal recognition accuracy in different Spanish accents.

There were two main ATA call scripts: one for screening and one for monitoring. The screening calls were for people who had no prior history of depression or who had been clear of a depression diagnosis for at least 6 months. The ATA collected information in four categories: (1) depressive symptoms; (2) pain; (3) self-management activities, including regular physical and fun activities; and (4) patient request to be contacted by a provider. PHQ-2, the first two items of the PHQ-9, were used for screening; if a patient score exceeded the cut-off of 3 points out of the possible 6 points, the ATA automatically asked the remaining PHQ-9 items. The monitoring calls were for depressed patients; the monitoring calls addressed all four categories and administered PHQ-9. If the patient had been prescribed an antidepressant, the call asked questions about medication adherence and side effects. If the patient was receiving psychotherapy, the call asked questions about problem-solving skills practice. Depending on the questions asked and patient response time, each ATA call lasted about 2 to 5 min. If a patient did not answer the call, the ATA system repeated the calling attempt multiple times a day for up to 1 week [52]. If a patient did not pick up the call within a week, the scheduled call was forfeited, and the patient was contacted again for the next scheduled call.

The telephone was selected as the communication platform because phones were the most accessible technology among safety-net patients at the time of the study. The calls were low intensity (ie, one call every month for monitoring or every 3 months for screening based on each patient’s depression condition) to balance information need and patient burden. Clinic officials have reported that patients who are depressed seem more likely to miss their scheduled visit appointments and often delay or forgo calling for help when symptoms fail to improve or worsen. The ATA system mitigated this dilemma by contacting patients rather than relying on them to initiate calls, proactively reaching patients and identifying their care needs. The TC model did accommodate patients who preferred a personal call over an automated call; in those cases, staff members made the calls according to the patient’s language and schedule preferences (25/366 or 6.8% of the patients made the request).

The patient-reported ATA data were tethered to the DMR, which in the TC model was enhanced by clinical decision support software for provider collaborative communication. The decision support software automatically generated task reminders and alerts based on the patient records in the DMR and the assessment data; the reminders and alerts prompted DMP providers to follow up with specific patients in need of care. For example, the automatically generated provider tasks in the DMR would remind a care manager to follow up with a patient who self-reported an antidepressant adherence issue, task a social worker to follow up with a patient with major depression symptoms, or task a nonclinical assistant to address patient callback requests. Task reminders included structured, radio-button lists of potential care management actions with the option of free text to support evidence-based practices and to ease providers’ documentation burden.

If a patient expressed an inclination toward self-harm or suicide (ie, responded to PHQ-9 question 9 with a score of 2=more than half the days or 3=nearly every day), the ATA system automatically initiated contact (via mobile phone SMS [short message service] text message and email) with an emergency response physician to get help for the patient. If the physician did not respond within 15 min, the ATA system initiated contact with the next physician on the emergency response team. This process repeated up to the fifth physician (the first three were psychiatrists and the last two were emergency medicine physicians) to ensure the patient received attention. During the study, the ATA was able to reach an emergency response physician in every instance.

#### Provider Training and Depression Treatment Protocol

DMP depression care in both SC and TC was based on the LACDHS depression care protocol and treatment guideline, which was developed by the DCAT team and described in the study design paper [33]. All SC and TC care providers were trained by an expert psychiatrist in the collaborative depression care model and adaptive treatment approach via one of three webinars (each approximately 2 hours). They were also offered training in PST via a 1-day (6-hour) workshop conducted by an academic psychologist and a social worker faculty member who are PST experts. UC providers were also invited to participate in these training opportunities, but they were not given time off from clinical duties to participate in the trainings.

While TC used technology to support providers for depression symptom and treatment adherence monitoring and to facilitate care coordination, SC providers monitored patients in the traditional way by calling patients and coordinating care among themselves. All patients in the SC and TC groups received support from a nurse care manager by telephone or in the clinic; in the TC group, patients also received the ongoing follow-up ATA calls in English or Spanish. If a patient in either group was confirmed for depression, weeks 1 to 8 of the depression care protocol included first-line treatment with antidepressant medication prescribed according to the protocol by the treating physician or nurse practitioner. If the patient refused medication, the care manager referred the patient to a social worker for six to eight PST sessions.

During weeks 9 to 12, the care manager would refer patients with a partial response (reduction in PHQ-9 scores) or nonresponse back to the treating physician or nurse practitioner, who would adjust antidepressant medication dosage (or encourage nonmedicated patients to begin medication) and the addition of PST. Patients with a full response (PHQ-9 score less than 8) received monthly treatment maintenance and relapse-prevention behavioral activation.

Consistent with the depression care protocol, patients with persistent PHQ-9 scores of 10 or higher were offered additional PST booster sessions; augmentation with low-dose trazodone, an antidepressant medication that also helps treat anxiety and insomnia; or referrals to specialty mental health care.

### Participant Eligibility and Recruitment

Textboxes 1 and 2 show the eligibility and ineligibility criteria for patients.

Every enrollee received a set of educational and community resource materials in Spanish or English.

The enrollment period was from April 2011 to May 2012. Patients with type 2 diabetes were identified for recruitment from the DMR database and clinic records. Patients provided verbal consent to bilingual research assistants during study eligibility screening.

**Eligibility criteria for patients.**

Eligibility criteria

- 18 years or older
- Had been diagnosed with type 2 diabetes
- Had a working phone number
- Spoke English or Spanish
- Could read and understand the consent form

**Ineligibility criteria for patients.**

Ineligibility criteria

- Patients with baseline possible suicidal ideation
- Patients with cognitive impairment
- Patients with alcohol abuse
- Patients with recent lithium or antipsychotic medication

**Table 2:** Primary outcome measures in the Diabetes-Depression Care-management Adoption Trial (DCAT), Los Angeles, 2011 to 2013. HBA_1c_: glycated hemoglobin; PHQ-9: 9-item Patient Health Questionnaire; PST: problem-solving therapy; SF-12: 12-item Short Form Survey.

Variables		Description
**Depression, measured at baseline and 6 months post intervention**		
	PHQ-9 [58]	Continuous variable assessing severity of depression. Scoring: PHQ-9 of 5-9=mild depression; PHQ-9 of 10-14=moderate depression; PHQ-9 of 15-19=major depression; PHQ-9 of 20-27=severe depression. For purposes of this study, PHQ-9 ≥10 indicated depression serious enough to consider pharmacologic or PST treatment.
	Depression remission	Dichotomous variable assessing effectiveness of treating patients with major depression. Depression remission defined as baseline PHQ-9 ≥10 and 6-month PHQ-9 ≤8 with a reduction ≥50%.
**Diabetes, measured at baseline and 6 months post intervention if not otherwise indicated**		
	HBA_1c_ value^a^	Continuous variable assessing severity of diabetes. HBA_1c_ value indicates average plasma glucose concentration over prolonged periods.
	HBA_1c_ tested^a^	Dichotomous variable assessing diabetes care process.
	Total cholesterol^a^	Continuous variable assessing cholesterol levels and severity of dyslipidemia
	Diabetes self-care [61]	Days per week of diabetes self-care. Treated as a continuous variable.
	Exercise	Days of participating in at least 30 min of exercise during previous week.
**Patient reported outcomes, measured at baseline and 6 months post intervention**		
	SF-12 physical score [62]	Continuous variables assessing functional health and well-being
	SF-12 mental score [62]	
	Sheehan Disability Scale [63,64]	Self-reported tool assessing functional impairment in work or school, social, and family life.
	Satisfaction with diabetes care	Five-level score assessing diabetes care satisfaction. Treated as a continuous variable.
	Satisfaction with care for emotional problems	Five-level score assessing mental health care satisfaction. Treated as a continuous variable.
	Satisfaction with care for emotional problems, baseline PHQ-9 ≥10	Five-level score assessing mental care satisfaction of patients with major depression. Treated as a continuous variable.

^a^The HBA_1c_ value, HBA_1c_ tested, and total cholesterol value were obtained from the LACDHS electronic medical record system. The measurement periods were within 3 months of baseline and 6-month post intervention. If more than one value was available, the values closest to the baseline and the 6-month follow-up period were chosen.

### Outcome Measures

Measures were taken at baseline and at 6, 12, and 18 months by independent English-Spanish bilingual interviewers. Primary outcomes included three depression outcomes, five diabetes care measures, and six patient-reported outcomes measuring physical and mental well-being, functional impairment, and satisfaction with care (see Table 2).

### Sample Size Calculation

The target sample size was based on power analysis for two primary outcomes: reduction of depressive symptoms (measured by PHQ-9 score) and depression remission. Power analyses were conducted using nQuery (Statistical Solutions Ltd, Boston MA) [65] to estimate effect sizes of the treatment with nonrandomized pre- and postintervention comparisons and longitudinal statistical approaches for repeated measures to compare trends in depression-related outcomes. The calculations assumed an alpha of .05, power of 0.80, attrition rates less than 20% at each 6-month follow-up assessment up to 18 months, and 25% to 30% depression prevalence among patients with diabetes [25]. For pre- and postintervention comparisons across all three program conditions, a sample size of approximately 500 in each study group would allow detection of a small effect size of 0.1.

### Statistical Analysis

All analyses were carried out according to the intention-to-treat rule consistent with standard practice in most clinical trials. The propensity score method has proved to be an effective approach to analyzing observational or quasi-experimental studies [66-70]. A propensity score is defined as the probability that a patient is likely to receive treatment or control given the patient’s baseline characteristics. Patients with the same propensity scores are like those in a randomized controlled trial.

The classical propensity score method is only applicable to two-way comparisons. Thus, we used a generalized propensity score (GPS) method designed for comparing two or more interventions versus one comparison group [71,72], wherein the GPS is defined as the conditional probability that a patient is likely to be in a specific group given this patient’s baseline characteristics. As recommended [72], a multinomial logistic regression was used to estimate GPSs. The model used study group as the dependent variable and the measured baseline characteristics shown in Table 3 (see “Results”) as the independent variables. We subsequently checked the distribution of the estimated GPSs because comparisons between groups are suspect if substantial separation occurs between study groups [72-74].

Comparative treatment effects were estimated by linear or logistic regression models that used outcomes at 6 months as the dependent variable and study group, care team, outcome variables at baseline, two of the three estimated GPSs, insulin use, HBA_1c_, age, gender, and preferred language as the independent variables. Regression that includes estimated GPSs as covariates has been shown to be an effective tool to adjust sample biases in observational or quasi-experimental studies [71,72]. Three care team variables were used to adjust for differences among providers. Two of the three estimated GPSs adjusted for imbalance in baseline characteristics. Insulin use, HBA_1c_, age, gender, and preferred language were included because their effects on outcomes were of clinical interest; and their inclusion is consistent with prior findings in behavioral and clinical factors associated with depression in patients with diabetes [75]. The coefficients of study group predicted comparative treatment effects while controlling for other covariates. All statistical analyses were conducted at 0.05 significance level (two-tailed) using SAS (SAS Institute Inc, Cary NC) software, version 9.3.

## Results

### Baseline Characteristics and Participant Flow

A total of 1704 patients were screened, of which 101 patients met the exclusion criteria, 128 patients refused to participate, 12 patients did not sign the Health Insurance Portability and Accountability Act agreement, and 57 patients were excluded after not completing the baseline assessment. Men had a significantly lower enrollment rate than women (84.0% [536/638] vs 89.02% [949/1066], respectively; *P*=.003), which was partly associated with poor alcohol use scores (4.9% [31/638] for men vs 0.56% [6/1066] for women).

**Figure 1:**
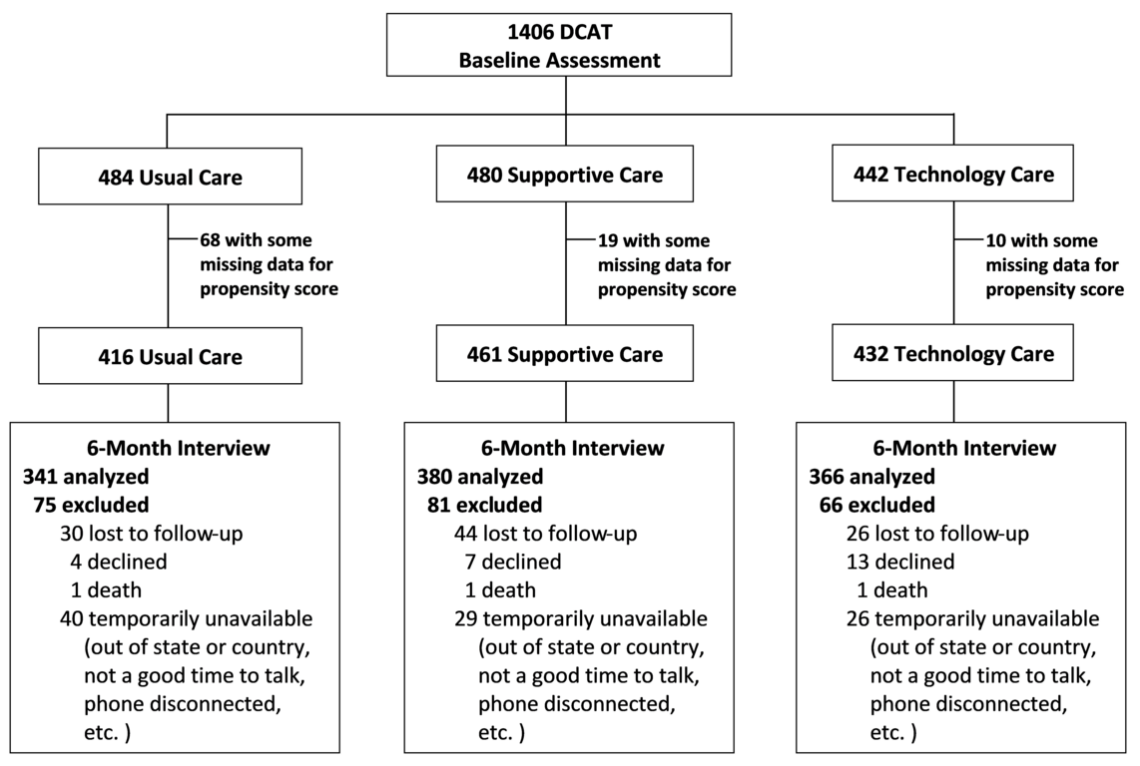
Consolidated Standards of Reporting Trials (CONSORT) diagram: participant flow of Diabetes-Depression Care-Management Adoption Trial (DCAT).

Among the 1406 patients enrolled in DCAT (484 in UC, 480 in SC, and 442 in TC), 1309 patients (416 in UC, 461 in SC, and 432 in TC) had complete data in the measures used in estimating the GPSs after interviews at baseline. As shown in Table 3, there were no significant differences in baseline depressive symptoms measured by the PHQ-9 score, anxiety symptoms measured by the Brief Symptom Inventory score, functional disability measured by the Sheehan Disability Scale (SDS), and the overall mental status measured by the 12-item Short Form Survey mental score. SC and TC patients had higher HBA_1c_ compared with UC patients because the SC and TC patients were enrolled from the DMP program designed for patients with severe diabetes. Other significant differences were diabetes self-care score and psychological stress measures (economic stress, number of stressors, sum of stress level, diabetes emotional burden, and diabetes regimen stress). The unbalanced samples were expected because of the quasi-experimental design. A Consolidated Standards of Reporting Trials diagram outlining participant flow is shown in Figure 1. See Multimedia Appendix 1 for comparison of baseline characteristics of samples included in versus excluded from the regression analysis. No significant differences were identified.

### Six-Month Outcomes

With the final sample size of 1087 (341 in UC, 380 in SC, and 366 in TC) to evaluate intervention effects, the study has the statistical power of 0.80 to detect an effect size of Cohen *d*=0.12, a small effect size. Regression analysis with GPS adjustment results regarding 6-month outcomes in DCAT are shown in Tables 4 and 5. The GPSs were estimated by the previously described multinomial logistic regression model. The distributions of the estimated GPSs across study groups were similar; thus, treatment effects can be predicted based on the estimated GPSs instead of actual group assignment.

Compared with UC, both SC and TC were significantly associated with decreased PHQ-9 scores (least squares estimate, LSE: UC=6.35, SC=5.05, TC=5.16; *P* value: SC vs UC=.02, TC vs UC=.02) and reduced prevalence of depression as measured by PHQ-9 ≥10 (SC vs UC: adjusted odds ratio, AOR=0.45, 95% CI 0.23-0.88, *P*=.02; TC vs UC: AOR=0.33, 95% CI 0.17-0.65, *P*=.007). Only TC was significantly associated with improved depression remission relative to UC (AOR=2.98, 95% CI 1.08-8.25, *P*=.04), although SC came close. There were no significant differences in depression outcomes between the SC and TC groups.

Regarding diabetes care measures, no significant differences existed between SC and UC. However, TC was significantly associated with reduced total cholesterol level (LSE: UC=176.40, TC=160.46; *P*=.01) and increased odds that the patient would have an HBA_1c_ test (TC vs UC: AOR=3.40, 95% CI 1.58-7.31, *P*<.001). The latter was positively correlated with depression remission (AOR=2.67, 95% CI 1.15-4.17, *P*=.004). There were no significant differences in diabetes care measures between the SC and TC groups.

**Table 3:** Descriptive of baseline measures used in estimating the generalized propensity scores. PHQ-9: 9-item Patient Health Questionnaire; SC: supported care; SF-12: 12-item Short Form Survey; TC: technology-facilitated care; UC: usual care.

Baseline characteristic	Usual care (n=416)^a^	Supported care (n=461)^a^	Technology-facilitated care (n=432)^a^	SC vs UC (*P* value)	TC vs UC (*P* value)	TC vs SC (*P* value)
Age in years, mean (SD)	55.15 (9.21)	51.92 (9.29)	52.63 (8.74)	<.001	<.001	.47
Female, n (%)	293 (70.4)	271 (58.8)	266 (61.6)	<.001	.02	.66
Latino, n (%)	389 (94.0)	386 (83.7)	390 (90.5)	<.001	.23	.003
Spanish as preferred language, n (%)	366 (88.0)	360 (78.1)	352 (81.5)	<.001	.03	.38
Body mass index, mean (SD)	32.55 (7.04)	32.73 (7.64)	33.11 (7.16)	.92	.50	.72
Less than high school education, n (%)	310 (74.5)	287 (62.3)	306 (70.8)	<.001	.47	.02
Unemployed, n (%)	275 (66.1)	30 (67.0)	286 (66.2)	.96	.99	.96
Economic distress^b^, mean (SD)	3.91 (2.44)	3.76 (1.98)	4.35 (2.10)	.57	.009	<.001
Number of stressors^c^, mean (SD)	2.16 (2.20)	2.57 (2.30)	2.54 (2.11)	.02	.03	.98
Sum of stress level^d^, mean (SD)	14.50 (16.23)	19.26 (19.49)	17.16 (16.87)	<.001	.07	.18
Predicted future health cost^e^, mean (SD)	6711.47 (3347.32)	6839.82 (3854.07)	6376.52 (3930.77)	.87	.39	.15
Age at onset of diabetes, mean (SD)	45.20 (10.52)	41.84 (10.19)	42.32 (9.84)	<.001	<.001	.76
Insulin use, n (%)	114 (27.4)	310 (67.2)	282 (65.3)	<.001	<.001	.80
SF-12 physical, mean (SD)	43.24 (11.19)	45.83 (10.91)	43.96 (10.89)	.002	.61	.03
SF-12 mental, mean (SD)	50.09 (12.12)	49.33 (14.16)	50.39 (12.44)	.66	.94	.45
Number of diabetes complications^f^, mean (SD)	0.71 (0.45)	0.73 (0.44)	0.65 (0.48)	.76	.15	.02
Whitty-9 diabetes symptoms scale^g^, mean (SD)	1.67 (0.63)	1.71 (0.63)	1.56 (0.53)	.54	.03	<.001
Diabetes emotional burden^h^, mean (SD)	2.75 (1.96)	3.69 (2.08)	2.53 (1.88)	<.001	.22	<.001
Diabetes regimen stress^h^, mean (SD)	2.61 (1.91)	3.61 (2.11)	2.40 (1.85)	<.001	.27	<.001
Diabetes self-care^i^, mean (SD)	4.04 (1.34)	4.76 (1.24)	4.23 (1.23)	<.001	.07	<.001
PHQ-9^j^, mean (SD)	6.55 (5.51)	6.80 (6.43)	6.44 (5.97)	.81	.96	.65
Brief Symptom Inventory^k^, mean (SD)	1.32 (3.02)	1.27 (3.24)	0.98 (2.72)	.96	.22	.32
Sheehan Disability Scale^l^, mean (SD)	2.19 (2.80)	2.10 (3.00)	2.06 (2.87)	.87	.78	.98
Dysthymia, n (%)	55 (13.2)	116 (25.2)	64 (14.8)	<.001	.81	<.001
Previous diagnosis of major depressive disorder, n (%)	23 (5.5)	75 (16.3)	17 (3.9)	<.001	.68	<.001
Chronic pain, n (%)	127 (30.5)	129 (28.0)	71 (16.4)	.65	<.001	<.001
Satisfaction with diabetes care, mean (SD)	4.61 (0.74)	4.81 (0.50)	4.67 (0.53)	<.001	.33	<.001
Satisfaction with care for emotional problems, mean (SD)	4.22 (0.99)	4.70 (0.63)	4.52 (0.66)	<.001	<.001	<.001
HBA_1c_ value, mean (SD)	8.37 (1.93)	9.57 (2.20)	9.73 (1.93)	<.001	<.001	.46

^a^Values are numbers (column percentage) for categorical variables and mean (SD) for continuous variables.

^b^Assessed by 12 general and health-related economic distresses, scored 0-12; higher scores indicate a higher level of economic distress.

^c^Assessed by 12 stressors related to work, family, social, and legal problems, scored 0-12; higher scores indicate a larger number of stressors.

^d^Assessed by 12 stressors related to work, family, social, and legal problems, each rated by level of stress from 0-10; therefore, total scores range from 0-120, with higher scores indicating a higher level of stress.

^e^Prediction of future health cost using the RxRisk model [76].

^f^Assessed by 7 diabetes complications: vision problems, loss of feeling in feet or legs, kidney problems, foot ulcer, amputation, sexual impairment, and heart attack, scored 0-7; higher scores indicate a larger number of diabetes complications.

^g^Assessed by the 9-item diabetes symptoms scale [77], scored 1-5; higher scores indicate more severe diabetes.

^h^Assessed by the 2-item Diabetes Distress Scale [78], scored 1-6; higher scores indicate a higher level of diabetes distress.

^i^Assessed by the Toobert Diabetes Selfcare Scale [61], scored 0-7; higher scores indicate better diabetes self-care.

^j^Assessed by the 9-item Patient Health Questionnaire [58], scored 0-27; higher scores indicate worse depressive symptoms.

^k^Assessed by the Brief Symptoms Inventory [79], scored 0-24; higher scores indicate worse anxiety.

^l^Assessed by the Sheehan Disability Scale [63,64], scored 0-30; higher scores indicate more significant functional impairment.

**Table 4:** Regression analysis of continuous 6-month outcomes adjusted for baseline characteristics and propensity scores in the Diabetes-Depression Care-management Adoption Trial (DCAT), Los Angeles, 2011 to 2013. Linear regression models are adjusted for care team, outcome variable at baseline, two of the three estimated generalized propensity scores, insulin use, glycated hemoglobin (HBA_1c_), age, gender, and preferred language. Least squares means and SE reported for continuous outcomes. LSE: least squares estimate; PHQ-9: 9-item Patient Health Questionnaire; SC: standard care; SF-12: 12-item Short Form Survey; TC: technology-facilitated care; UC: usual care.

Continuous outcome	Usual care (n=341), LSE (SE)	Supported care (n=380), LSE (SE)	Technology-facilitated care (n=366), LSE (SE)	SC vs UC (*P* value)	TC vs UC (*P* value)	TC vs SC (*P* value)
PHQ-9	6.35 (0.49)	5.05 (0.47)	5.16 (0.48)	.02	.02	.81
HBA_1c_ value	7.95 (0.17)	7.79 (0.16)	8.05 (0.16)	.41	.57	.10
Total cholesterol	176.40 (5.27)	166.90 (4.96)	160.46 (5.04)	.12	.01	.16
Diabetes self-care	4.66 (0.13)	4.70 (0.12)	4.78 (0.12)	.80	.38	.52
Exercise	4.73 (0.28)	4.90 (0.26)	4.86 (0.27)	.59	.66	.88
SF-12 physical score	42.99 (0.97)	42.46 (0.95)	41.87 (0.95)	.63	.27	.55
SF-12 mental score	48.38 (1.04)	50.07 (1.01)	49.87 (1.02)	.16	.17	.85
Sheehan Disability Scale	3.21 (0.26)	2.61 (0.25)	2.59 (0.25)	.04	.03	.95
Satisfaction with diabetes care	4.01 (0.09)	4.15 (0.09)	4.20 (0.09)	.17	.05	.58
Satisfaction with care for emotional problems	3.25 (0.10)	3.64 (0.10)	3.46 (0.10)	.01	.07	.06
Satisfaction with care for emotional problems, among patients with baseline PHQ-9 ≥10	3.20 (0.22)	3.58 (0.21)	3.70 (0.21)	.16	.05	.56

**Table 5:** Regression analysis of binary 6-month outcomes adjusted for baseline characteristics and propensity scores in the DCAT, Los Angeles, 2011-2013. Logistic regression models are adjusted for care team, outcome variable at baseline, two of the three estimated generalized propensity scores, insulin use, glycated hemoglobin (HBA_1c_), age, gender, and preferred language. Adjusted odds ratio (AOR) and 95% CIs reported for binary outcomes. PHQ-9: 9-item Patient Health Questionnaire

Binary outcome	Supported care vs usual care		Technology-facilitated care vs usual care		Technology-facilitated care vs supported care	
	AOR (95% CI)	*P* value	AOR (95% CI)	*P* value	AOR (95% CI)	*P* value
PHQ-9≥10	0.45 (0.23-0.88)	.02	0.33 (0.17-0.65)	.007	0.75 (0.39-1.41)	.37
Depression remission	2.86 (0.98-8.40)	.06	2.98 (1.08-8.25)	.04	1.04 (0.47-2.31)	.92
HBA_1c_ tested^a^	1.82 (0.89-3.71)	.10	3.40 (1.58-7.31)	<.001	1.87 (0.82-4.27)	.14

^a^Adjusted relative risk for HBA_1c_ tested, supported care vs usual care=1.13 (0.97-1.23), technology-facilitated care vs usual care=1.22 (1.10-1.29), technology-facilitated care vs supported care=1.12 (0.95-1.21).

Both SC and TC were significantly associated with improved SDS scores relative to UC (LSE: UC=3.21, SC=2.61, TC=2.59; *P* value: SC vs UC=.04, TC vs UC=.03). SC was significantly associated with improved satisfaction with care for emotional problems compared with UC (LSE: UC=3.25, SC=3.64; *P*=.01), but only TC was significantly associated with improved satisfaction with diabetes care (LSE: UC=4.01, TC=4.20; *P*=.05) and satisfaction with care for emotional problems among patients with depression, as measured by PHQ-9 ≥10 at baseline (LSE: UC=3.20, TC=3.70; *P*=.05). There were no significant differences in patient-reported outcomes between the SC and TC groups.

## Discussion

### Principal Findings

Analysis of 6-month DCAT outcomes revealed that both the TC and SC groups were significantly associated with better outcomes compared with UC in terms of depressive symptoms reduction. Using the PHQ-9 score, which ranges from 0 to 27, the 1.3 (SC group) and 1.2 (TC group) points of improvements compared with the UC group are clinically meaningful given that the baseline PHQ-9 score is only about 6.5 points. The magnitude of improvements is consistent with a recent collaborative depression care study that included both depressed and nondepressed patients [80]. This finding supports the hypothesis that the two intervention groups would be associated with better depression care outcomes.

It was not surprising to find the ATA technology did not improve depression outcomes of the TC group over the SC group in this case because it was designed to facilitate the adoption of collaborative depression care rather than direct clinical intervention with patients. Clinically, DMP care teams in both groups were trained in and practiced the LACDHS depression protocol and treatment guideline. The SC DMP providers monitored patients using traditional mechanisms (specifically by calling patients), and they coordinated care among themselves; therefore, the study-related depression care resulted in additional new workload for the SC providers. For the TC care team, the technology, albeit at a low-intensity of contact, helped alleviate the workload for depression symptom and treatment adherence monitoring.

The ATA technology also prompted providers to follow up and alerted emergency responders to immediately contact patients with suicidal ideation. That could be the reason why only the TC group was significantly associated with depression remission and increased patient satisfaction among depressed patients. The results are encouraging that a well-designed technology can be an effective aide to the adoption of collaborative depression care. Full justification of the TC model via a complete cost-effectiveness analysis is presented elsewhere [56]. The cost-effectiveness analysis revealed that the intervention models improved quality-adjusted life years, depression-free days, and medical costs. The TC model was cost-effective compared with SC and cost-saving compared with UC. The 6-month and cost-effectiveness results suggest the TC model is promising in facilitating better and cost-effective care for depressed patients.

Moreover, only the TC model was significantly associated with improved diabetes care processes, indicated by reduced total cholesterol level and increased odds that the patient would have an HBA_1c_ test. One possible explanation for these improvements in diabetes care is that as depressive symptoms are increasingly monitored and timelier addressed, patients may become more willing to take active care of their diabetes; this explanation is supported by the significant correlation between the odds of having an HBA_1c_ test and the improvement in depression remission. Another possible explanation is that providers may address patients’ diabetes care needs in addition to depression care needs when they respond to the task reminders generated by the technology.

Compared with the UC group, both the SC and TC models were significantly associated with better improvement in 6-month patient-reported functioning in family, work, and social life, as measured by the 3-item SDS. The 0.6 points of improvements in SC and TC groups compared with the UC group are meaningful as most patients at baseline had only minimal functional impairment (average baseline SDS score was 2.1 points; SDS score >6 indicates functional disability [63,64]), which implies that the room for improvement is small. This finding suggests that the two enhanced care delivery models not only improved depressive symptoms but also translated such symptom improvement into better perceived life functioning.

While the TC model delivered positive results, and most patients in the TC groups reported high acceptance of the ICT tested in DCAT [51], there is significant room for improvement in using the ATA technology. Specifically, only half of the scheduled calls were answered successfully because of phone connectivity issues or lack of time for the patient to answer the calls [50,52]. One challenge may have been that during the study, most patients in LACDHS did not use cellphones. Now that cellphones are more readily available, attention should be turned to other ICT (such as SMS text messages and smartphone apps) to improve patient contact and to capture patient-reported outcomes. Such technology has greater portability and versatility, may extend the ATA capabilities in reaching and engaging patients, may potentially increase the model effectiveness, and may reduce costs.

Researchers can expect unpredictable consequences after making changes (such as the DCAT implementation) in complex systems such as LACDHS. As discussed in another DCAT study [53], two particularly important implications that emerged were the strengthened role of social workers (in both SC and TC) and the importance of suicide-alert responders (in TC). Every DMP site had a colocated clinical social worker, an evidence-based method of improving quality of depression care [81,82]. The clinical social workers were an underutilized resource before the study; during the DCAT trial, they proved instrumental in adopting depression care. Furthermore, the suicide-alert responders appeared to play a much larger role than anticipated. Providers facing typical barriers in mental health care (including lack of familiarity with guidelines, lack of self-efficacy, and lack of outcome expectancy [83]) were reassured by the availability of an organizational resource for the patient to fall back on in the “worst case scenario,” namely severe suicidal ideation. Taken together with the strengthened role of the social worker, the interventions seemed to have leveraged the available mental health resources into a more cohesive, integrated model of mental health care in a primary setting. In other words, the SC and TC models used existing diabetes disease management teams and leveraged available mental health resources to implement depression symptom monitoring and treatment protocols, provider collaborative communication, and patient relapse prevention.

In summary, the 6-month DCAT findings suggest that both the TC and SC delivery models are significantly associated with improved depression outcomes and life functioning, and that the TC model offers additional promise in terms of improved depression remission, diabetes care processes, and patient satisfaction. Given the rapid rise of diabetes during the past several decades—especially among low-income, minority populations—and the immense opportunity to improve diabetes-related measures and outcomes, a growing number of health plans and health care organizations are trying to manage their diabetes population through disease management programs. The SC and TC models demonstrated that an important and valuable way to support providers is to add evidence-based collaborative depression care and facilitate adoption of ICT in diabetes DMPs designed to reduce disparities in commonly comorbid diabetes and depression care. When enhanced by ICT, DMPs may be able to greatly improve overall care, cost, and effectiveness of health care delivery for underserved patients. DCAT SC and TC models improved diabetes and depression outcomes in the second largest US safety-net health system; other resource-constrained programs may replicate these models to improve comorbid diabetes and depression outcomes.

### Limitations

The main limitation of this study is its employment of a quasi-experimental design, which introduces bias because of the differences in both patient characteristics and care teams at each facility. To mitigate the bias, in the regression analysis we adjusted for patient differences through propensity scores and the assignment of the six care teams at eight facilities (each facility was staffed by only one team; two of the six teams served two facilities). Although care facilities were matched by geographic location and patient sociodemographics among the three study groups, the quality of care can vary from facility to facility; therefore, the regression analysis included a check in which care team assignments were replaced for each facility. This analysis did not change the direction and significance of intervention effects; however, the adjustment may not be sufficient. Differences in the facilities, the DMP care teams, and the unmeasured patient sociodemographics, diabetes and comorbid conditions, and psychological stress measures may differentiate diabetes and depression care needs and outcomes [84]. Providers should consider these differences when applying the technology.

Another limitation may be the predominantly Latino sample, which raises concern about the generalizability of findings. Applying the DCAT TC model to other groups should be done cautiously and with further evaluation.

The third limitation is the focus in this paper on clinical outcomes. However, the DCAT TC model was designed to accelerate the adoption of evidence-based collaborative care to improve the overall care process. Full analyses of 18-month clinical outcomes and the cost-effectiveness of the TC model will be reported elsewhere.

The fourth limitation is lack of data to understand the practical mechanism by which the SC DMP and the technology-enhanced TC DMP led to improvements in depression. Possible improvement mechanisms for future research include better treatment initiation or adjustment, receipt of PST, greater patient adherence, or referrals and visits to other mental health providers.

### Comparison With Prior Work

DCAT adds to the growing number of telehealth studies that are employing technology to improve depression care in primary care settings for patients with chronic diseases [85-92]. A key strength of the DCAT TC model over earlier studies is that it used automated calls, which reduces provider depression monitoring workload and allows more time for clinical encounters such as timely adjustment of treatment. The TC model is especially effective in a resource-constrained environment such as safety-net care systems, improving care for predominantly minority and low-income patients.

Applying automated remote monitoring ICT, electronic clinical decision support, and even artificial intelligence to facilitate chronic disease management is an emerging research topic. Prior studies revealed that ATA is valid in conducting depression screening and suggested the technology can be incorporated into the care management model [93,94]. However, evidence is limited regarding the comparative effectiveness of the technology. Kroenke et al [95] tested ATA with care management for pain and depression in patients with cancer; Ratanawongsa et al [96] and Handley et al [97] tested ATA-facilitated diabetes management for low-income Medicaid and safety-net patients. The DCAT study uniquely addressed depression care for patients with type 2 diabetes in a safety-net setting where comorbidity of the two diseases is common and where many barriers such as culture diversity, financial stress, and limited provider resources impede the adoption of evidence-based depression interventions. Results from DCAT are consistent with prior studies [91-97] and support ATA as a promising technology to facilitate care management, even for sensitive conditions such as depression, for diverse populations and in primary care settings.

### Conclusions

Both SC and TC models are associated with improved 6-month depression outcomes and reduced functional disability among adult patients with type 2 diabetes. However, the TC model is more likely to achieve greater improvements in depression remission, as well as measures of patient satisfaction and diabetes care quality. This paper provides encouraging evidence that a well-designed automated ICT system is an effective facilitator that can support delivery of evidence-based collaborative depression care to patients with type 2 diabetes in a resource-constrained urban safety-net primary care setting. This is a promising solution to reduce health disparities, improve patient experience of care, and improve the health of low-income, minority populations.

## Appendix

Multimedia Appendix 1 Comparison of baseline characteristics of included vs excluded samples for the regression analysis.

## Abbreviations

HBA _1c_: glycated hemoglobin
AOR: adjusted odds ratio
ATA: automatic telephone assessment
DCAT: Diabetes-Depression Care-Management Adoption Trial
DMP: disease management program
DMR: disease management registry
GPS: generalized propensity score
ICT: information and communication technology
LACDHS: Los Angeles County Department of Health Services
LSE: least squares estimate
OR: odds ratio
PCP: primary care physician
PHQ-9: 9-item Patient Health Questionnaire
PST: problem-solving therapy
SC: supported care
SDS: Sheehan Disability Scale
SF-12: 12-item Short Form Survey
TC: technology-facilitated care
UC: usual care
