# Supplementary material for: Comparative Effectiveness of a Technology-Facilitated Depression Care Management Model in Safety-Net Primary Care Patients With Type 2 Diabetes: 6-Month Outcomes of a Large Clinical Trial
Source: J Med Internet Res. 2018 Apr 23;20(4):e147. doi: 10.2196/jmir.7692 (PMC5938593; doi:10.2196/jmir.7692)
Supplement: Multimedia Appendix 1 [file jmir_v20i4e147_app1.pdf]

Appendix. Comparison of baseline characteristics of included vs excluded samples for the regression analysis.

Baseline characteristic	UC			SC			TC		
	Included	Excluded	*P*	Included	Excluded	*P*	Included	Excluded	*P*
	(n=341)	(n=75)		(n=380)	(n=81)		(n=366)	(n=66)	
Age	55.58 (8.75)	53.20 (10.91)	.08	51.93 (9.21)	51.84 (9.68)	.94	52.46 (8.51)	53.56 (9.93)	.40
Female	241 (70.7)	52 (69.3)	.93	221 (58.2)	50 (61.7)	.64	230 (62.8)	36 (54.5)	.26
Latino	320 (94.4)	69 (92.0)	.60	318 (83.7)	68 (84.0)	.99	332 (91.0)	58 (87.9)	.58
Spanish as preferred language	302 (88.6)	64 (85.3)	.56	300 (78.9)	60 (74.1)	.42	305 (83.3)	47 (71.2)	.06
Body mass index	32.33 (6.84)	33.56 (7.83)	.21	32.84 (7.52)	32.23 (8.20)	.53	33.37 (7.10)	31.68 (7.40)	.09
Less than high school education	260 (76.2)	50 (66.7)	.11	239 (62.9)	48 (59.3)	.63	259 (70.8)	47 (71.2)	.99
Unemployed	221 (64.8)	54 (72.0)	.29	258 (67.9)	51 (63.0)	.47	242 (66.1)	44 (66.7)	.99
Economic distress[b]	3.91 (2.47)	3.92 (2.31)	.97	3.73 (1.98)	3.91 (1.99)	.46	4.33 (2.11)	4.47 (2.02)	.60
Number of stressors[c]	2.23 (2.22)	1.83 (2.13)	.15	2.59 (2.35)	2.49 (2.05)	.72	2.52 (2.07)	2.67 (2.34)	.63
Sum of stress level[d]	15.07 (16.39)	11.93 (15.34)	.12	19.43 (19.84)	18.46 (17.87)	.66	17.17 (16.88)	17.12 (16.94)	.98
Predicted future health cost[e]	6725.04 (3414.20)	6649.79 (3045.27)	.85	6808.22 (3583.73)	6988.09 (4953.62)	.76	6449.14 (3993.46)	5973.83 (3564.43)	.33
Age at onset of diabetes	45.71 (10.30)	42.88 (11.27)	.06	41.91 (10.23)	41.51 (10.07)	.75	42.29 (9.56)	42.52 (11.37)	.88
Insulin use	96 (28.2)	18 (24.0)	.56	254 (66.8)	56 (69.1)	.79	241 (65.8)	41 (62.1)	.66
SF-12 physical	43.21 (11.31)	43.40 (10.72)	.89	45.97 (10.91)	45.16 (11.00)	.55	43.85 (11.03)	44.56 (10.11)	.61
SF-12 mental	50.20 (12.02)	49.62 (12.64)	.72	49.60 (13.84)	48.09 (15.58)	.42	50.53 (12.29)	49.60 (13.31)	.60
Number of diabetes complications[f]	0.73 (0.44)	0.63 (0.49)	.09	0.74 (0.44)	0.68 (0.47)	0.25	0.66 (0.48)	0.64 (0.48)	.77
Whitty-9 diabetes symptoms scale[g]	1.66 (0.65)	1.69 (0.55)	.69	1.73 (0.65)	1.63 (0.53)	.15	1.57 (0.53)	1.53 (0.52)	.52
Diabetes emotional burden[h]	2.79 (2.00)	2.61 (1.79)	.46	3.76 (2.07)	3.37 (2.10)	.14	2.53 (1.90)	2.52 (1.80)	.95
Diabetes regimen stress[h]	2.64 (1.93)	2.48 (1.83)	.50	3.67 (2.12)	3.30 (2.08)	.14	2.41 (1.85)	2.33 (1.86)	.75
Diabetes self-care[i]	4.07 (1.29)	3.89 (1.52)	.33	4.76 (1.24)	4.72 (1.24)	.79	4.29 (1.21)	3.99 (1.30)	.06

PHQ-9[j]	6.34 (5.52)	7.49 (5.38)	.10	6.73 (6.29)	7.10 (7.05)	.67	6.42 (5.85)	6.55 (6.65)	.89
Brief Symptom Inventory[k]	1.28 (2.87)	1.52 (3.60)	.59	1.21 (3.23)	1.56 (3.27)	.39	0.98 (2.80)	1.00 (2.27)	.94
Sheehan Disability Scale[l]	2.14 (2.84)	2.44 (2.65)	.39	2.10 (2.97)	2.07 (3.14)	.92	2.03 (2.86)	2.23 (2.96)	.61
Dysthymia	45 (13.2)	10 (13.3)	.99	94 (24.7)	22 (27.2)	.75	54 (14.8)	10 (15.2)	.99
Previous diagnosis of major depressive disorder	21 (6.2)	2 (2.7)	.36	61 (16.1)	14 (17.3)	.91	13 (3.6)	4 (6.1)	.53
Chronic pain	103 (30.2)	24 (32.0)	.87	106 (27.9)	23 (28.4)	.99	61 (16.7)	10 (15.2)	.90
Satisfaction with diabetes care	4.58 (0.77)	4.72 (0.58)	.09	4.83 (0.45)	4.72 (0.68)	.14	4.66 (0.54)	4.71 (0.49)	.42
Satisfaction with care for emotional problems	4.24 (0.99)	4.15 (1.03)	.51	4.73 (0.59)	4.57 (0.77)	.08	4.53 (0.64)	4.43 (0.73)	.30
A1c value	8.36 (1.94)	8.41 (1.90)	.84	9.52 (2.16)	9.80 (2.38)	.32	9.72 (1.92)	9.77 (2.00)	.87

[a]A1c: glycated hemoglobin; PHQ-9: 9-item Patient Health Questionnaire; SF-12: Short Form-12 Health Survey.

[b]Assessed by 12 general and health-related economic distresses, scored 0-12; higher scores indicate a higher level of economic distress.

[c]Assessed by 12 stressors related to work, family, social, and legal problems, scored 0-12; higher scores indicate a larger number of stressors.

[d]Assessed by 12 stressors related to work, family, social, and legal problems, each rated by level of stress from 0-10; therefore, total scores range from 0-120, with higher scores indicating a higher level of stress.

[e]Prediction of future health cost using the RxRisk model [76].

[f]Assessed by 7 diabetes complications: vision problems, loss of feeling in feet or legs, kidney problems, foot ulcer, amputation, sexual impairment, and heart attack, scored 0-7; higher scores indicate a larger number of diabetes complications.

[g]Assessed by the 9-item diabetes symptoms scale [77], scored 1-5; higher scores indicate more severe diabetes.

[h]Assessed by the 2-item Diabetes Distress Scale [78], scored 1-6; higher scores indicate a higher level of diabetes distress.

[i]Assessed by the Toobert Diabetes Selfcare Scale [61], scored 0-7; higher scores indicate better diabetes self-care.

[j]Assessed by the 9-item Patient Health Questionnaire [58], scored 0-27; higher scores indicate worse depressive symptoms.

[k]Assessed by the Brief Symptoms Inventory [79], scored 0-24; higher scores indicate worse anxiety.

[l]Assessed by the Sheehan Disability Scale [63,64], scored 0-30; higher scores indicate more significant functional impairment.
